# Insulin in the Medical Management of Postprandial Hypoglycemia in a Patient with Type 2 Diabetes after Gastric Bypass Surgery

**DOI:** 10.1155/2012/427565

**Published:** 2012-06-28

**Authors:** Jennifer Leung Schoenberger, Chung-Kay Koh, Tiffany Hor, David Baldwin, Arati Reddy, Lara Rondinelli-Hamilton

**Affiliations:** Section of Endocrinology, Rush University Medical Center, Chicago, IL 60612, USA

## Abstract

*Objective*. We evaluated a 47-year-old woman with a history of type 2 diabetes and severe obesity who developed postprandial hypoglycemia after undergoing Roux-en-Y gastric bypass surgery and losing 60% of her total body weight. We studied her insulin secretion and blood glucose dynamics and were able to tailor a therapeutic regimen involving insulin that eliminated episodes of hypoglycemia. *Methods*. We studied blood glucose levels during a prolonged fast, performed continuous glucose monitoring studies using a subcutaneous glucose sensor, and evaluated regional pancreatic insulin secretion using selective arterial calcium stimulation. *Results*. Continuous glucose monitoring revealed that the patient had early (1-2 hr) postprandial hyperglycemia followed by late (3-4 hr) postprandial hypoglycemia. Biochemical studies confirmed endogenous pancreatogenous insulin secretion as the cause of episodic hypoglycemia, but imaging studies and selective arterial calcium stimulation failed to localize an insulinoma. The patient was treated with preprandial doses of insulin aspart in order to attenuate the early postprandial hyperglycemia, and the late hypoglycemic episodes were avoided. *Conclusion*. We describe an interesting and novel nonsurgical approach to the prevention of postprandial hypoglycemia in a patient with noninsulinoma pancreatogenous hypoglycemia after gastric bypass.

## 1. Introduction

Postprandial hypoglycemia is recognized as a complication of Roux-en-Y gastric bypass surgery for obesity. After surgery, patients may experience postprandial dizziness, diaphoresis, and weakness, a phenomenon known as the dumping syndrome [1, 2]. This is hypothesized to be due to the rapid transit of hyperosmolar gastric contents into the jejunal lumen, causing a shift of fluid out of the intravascular space. Additionally, in occasional patients, this may stimulate an exaggerated secretion of insulin, leading to postprandial hypoglycemia and associated signs and symptoms of neuroglycopenia. In the past, the management of these patients has focused on dietary modification, *α*-glucosidase inhibition (acarbose), and somatostatin analogs [2–5]. The study by Service et al. of six such patients highlighted the effectiveness of partial pancreatectomy in their management, as well as the presence of diffuse nesidioblastosis as the primary histopathological finding. This represents an important subtype of pancreatogenous hyperinsulinemic hypoglycemia that is not caused by a typical single insulinoma [6]. 

We encountered a patient who developed postprandial hypoglycemia after gastric bypass surgery. Upon study we observed that her episodes of hypoglycemia were always preceded first by postprandial hyperglycemia, and this suggested a novel approach to her medical therapy. 

## 2. Case Presentation

The patient is a 47-year-old woman who complained of postprandial hypoglycemia for the past 7 months. She reported frequent capillary blood glucose (BG) readings as low as 24 mg/dL occurring 3-4 hours after meals. Hypoglycemia was accompanied by sweating, tunnel vision, and occasionally by seizures. Her symptoms resolved promptly after oral glucose or an injection of glucagon. She had a longstanding history of type 2 diabetes mellitus treated with subcutaneous insulin. She developed severe obesity and 3 years earlier had undergone Roux-en-Y gastric bypass surgery. Subsequently she lost 200 pounds and required much less insulin. However, 9 months after gastric bypass, she began experiencing frequent episodes of hypoglycemia, which persisted despite discontinuation of insulin and modification of her diet. She was taking no medications when she presented to us for evaluation 3 years after surgery. 

On physical exam, blood pressure was 120/74 and heart rate was 60 beats/min. Her weight had decreased to 116 pounds. She had a large well-healed incision; otherwise her examination was unremarkable. 

**Figure 1:**
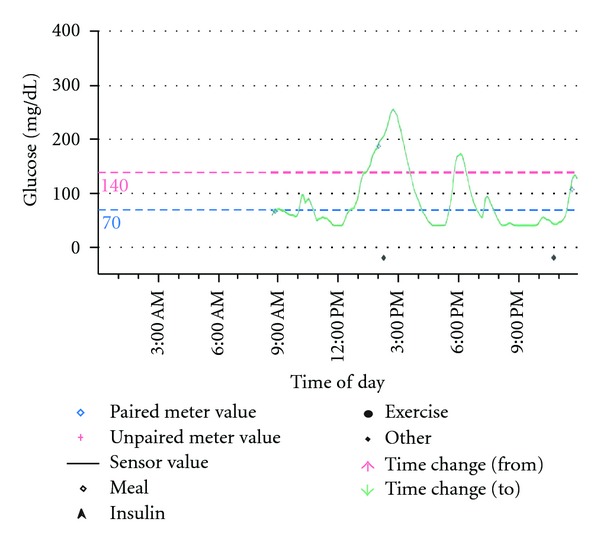
Continuous glucose monitoring sensor tracing prior to insulin dosing with meals. The patient had several episodes of hypoglycemia <50 mg/dL occurring 3-4 hours after hyperglycemia following a meal. Not all meals may have been documented accurately.

Initially, the patient underwent a monitored 36-hour fast. However, the lowest serum glucose was only 63 mg/dL, and she was asymptomatic. We then performed continuous glucose monitoring for 72 hours with a Medtronic sensor as an outpatient and discovered a pattern of hyperglycemia (BG 180–280 mg/dL) occurring 1-2 hours after a meal, followed by symptomatic hypoglycemia with BG <50 mg/dL occurring 3-4 hours thereafter (Figure 1). Further evaluation 3 hours after a mixed meal revealed serum glucose 40 mg/dL, serum insulin 3.0 *μ*U/mL, serum c-peptide 2.1 ng/mL, serum proinsulin 13 pmol/L, serum beta-hydroxybutyrate 0.1 mmol/L, and a negative serum screen for sulfonylureas. CT scan of the abdomen with contrast was normal. However, abdominal arteriography revealed a vague area of hypervascularity in the tail of the pancreas. Due to this finding and the severity of her symptoms, she underwent selective arterial calcium stimulation with hepatic venous sampling for insulin as described by Doppman et al. [7]. This test did not localize a region of insulin hypersecretion, and thus was not consistent with a diagnosis of insulinoma (Table 1).

**Figure 2:**
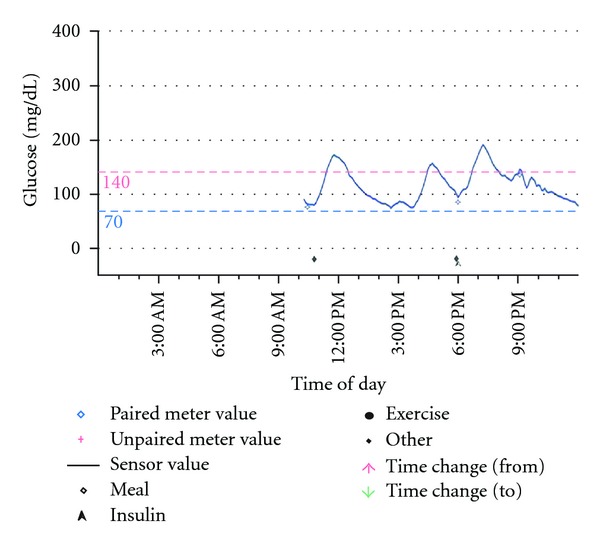
Continuous glucose monitoring sensor tracing after insulin dosing with meals. The degree of hyperglycemia following meals was improved after treatment with prandial insulin, and the patient no longer had any further episodes of hypoglycemia.

Our initial therapeutic approach to the patient was to try multiple small meals with low carbohydrate content. However, she continued to have episodic hypoglycemia. Subsequently, the patient underwent a trial of acarbose 25 mg with each meal. Hypoglycemia appeared to improve; however she developed intolerable diarrhea and was not willing to continue after 3 days. We then hypothesized that if exogenous mealtime insulin was provided with a subcutaneous rapid-acting insulin analog, the postprandial elevation in BG could be attenuated or eliminated, and the subsequent episode of hypoglycemia at 3-4 hours after meal could be prevented. We began insulin aspart at an initial dose of 1 unit per 30 grams of carbohydrate taken 10 minutes before each meal. The patient tested capillary BG before meals and 2 hours after meals. The dose of aspart was titrated until 2-hour postmeal readings were <180 mg/dL. We noted that she experienced no further episodes of postprandial hypoglycemia and remain symptom-free on this regimen. We repeated a continuous glucose monitoring study and confirmed that hyperglycemic excursions at 2 hours after meal were improved and hypoglycemic excursions at 3-4 hours after meal had resolved (Figure 2). 

## 3. Discussion

Because postprandial hypoglycemia is seen commonly after bariatric surgery, we initially attempted a conservative approach with dietary modification for presumed dumping syndrome, but this failed in our patient [2]. We then tried acarbose, an alpha-glucosidase inhibitor that interferes with carbohydrate absorption, which resolved the patient's postprandial symptoms and hypoglycemia, but she was unable to tolerate it due to gastrointestinal side effects, which are commonly seen with this medication [3]. We did not consider octreotide, a somatostatin analogue, which inhibits the release of insulin and many gastrointestinal hormones like glucagon-like peptide (GLP-1), due to its cost and significant adverse side effects including steatorrhea [4, 5]. Lastly, we did not want to resort to surgical reintervention or continuous enteral feeding, which are sometimes considered in patients refractory to conservative medical therapy. 

We therefore initiated prandial aspart insulin, hoping to blunt the postprandial glucose peak and excessive insulin surge and thus prevent the resultant hypoglycemia. She reported no further episodes of hypoglycemia, which was confirmed on 72-hour continuous glucose monitoring with and without prandial insulin. It has been postulated that this culprit insulin surge may stem from exaggerated GLP-1 seen in postgastric bypass [6, 8, 9]. It is unclear why her severe hypoglycemia only started 9 months after gastric surgery. Perhaps her dramatic weight loss improved her insulin sensitivity to the point that she could not accommodate a mistiming of insulin secretion and rapid carbohydrate passage due to gastric bypass. It has also been suggested that changes in beta cells postgastric bypass may lead to this inappropriate insulin secretion, though this was refuted by Meier et al. [10].

Nonetheless, this is the first report of which we are aware in which insulin was paradoxically used to prevent postprandial hypoglycemia. It is possible that the patient's history of type 2 diabetes mellitus and insulin resistance helped to prevent hypoglycemia with the administration of aspart insulin. However, as with most gastric bypass patients with preexisting diabetes, her insulin sensitivity tremendously improved after gastric bypass to the point where she no longer required treatment with insulin. We realized that while insulin therapy has the benefit of avoiding the gastrointestinal side effects of acarbose and octreotide, as well as the cost of the latter, the well-described side effect of weight gain with insulin may be a downfall as these patients underwent surgery to lose weight in the first place.

We also want to point out the possibility of other causes of endogenous hyperinsulinemic hypoglycemia in those who have undergone gastric bypass surgery, particularly insulinoma and noninsulinoma pancreatogenous hypoglycemia syndrome (NIPHS). Because hypoglycemia can have many detrimental consequences, including altered mental status, syncope, seizure, and even permanent neurologic damage and death, we find it essential that the correct diagnosis is made and not missed, as this will dictate treatment. If a patient with endogenous hyperinsulinemic hypoglycemia has persistent neuroglycopenia and hypoglycemia refractory to conventional treatment for dumping syndrome, further investigation including abdominal imaging and selective arterial calcium stimulation is required to confidently exclude insulinoma or NIPHS. Zagury et al. reported a case in which a patient's hypoglycemia status after gastric bypass was assumed to be due to dumping syndrome but was later found to be due to insulinoma [11].

In summary, we reviewed different treatment options for postprandial hypoglycemia in dumping syndrome and report the novel use of aspart insulin as an alternative treatment in those who fail, are unable to tolerate, or want to avoid the other proposed treatment modalities.

## Figures and Tables

**Table 1:** Results of hepatic venous insulin levels after selective arterial calcium stimulation.

Site of calcium injection	Hepatic venous insulin level (*μ*U/mL)			
	0 min	30 min	60 min	120 min
Superior mesenteric artery	4.9	5.8	6.0	4.9
Gastroduodenal artery	7.3	5.0	3.8	3.4
Splenic artery	2.9	3.8	4.0	3.6
